# Efficacy of lisdexamfetamine dimesylate in adults with attention-deficit/hyperactivity disorder previously treated with amphetamines: analyses from a randomized, double-blind, multicenter, placebo-controlled titration study

**DOI:** 10.1186/2050-6511-13-18

**Published:** 2012-12-19

**Authors:** Thomas Babcock, Bryan Dirks, Ben Adeyi, Brian Scheckner

**Affiliations:** 1 Shire Development LLC, 725 Chesterbrook Blvd, Wayne, PA, 19087, USA

**Keywords:** Lisdexamfetamine dimesylate, LDX, Amphetamines, Attention-deficit hyperactivity disorder, ADHD, Adult, Switching treatment

## Abstract

**Background:**

To examine the efficacy of lisdexamfetamine dimesylate (LDX) in adults with attention-deficit/hyperactivity disorder (ADHD) who remained symptomatic (ADHD Rating Scale IV [ADHD-RS-IV] total score >18) on amphetamine (AMPH) therapy (mixed AMPH salts and/or d-AMPH formulations) prior to enrollment in a 4-week placebo-controlled LDX trial vs the overall study population. In these post hoc analyses from a multicenter, randomized, double-blind, forced-dose titration study, clinical efficacy of LDX (30-70 mg/d) in adults with ADHD receiving AMPH treatment at screening vs the overall study population was evaluated. ADHD symptoms were assessed using the ADHD-RS-IV with adult prompts at screening, baseline (after prior treatment washout), and endpoint. Safety assessments included treatment-emergent adverse events (TEAEs), vital signs, laboratory findings, and electrocardiogram.

**Results:**

Of 414 participants (62, placebo; 352, LDX) included in the overall study population, 41 were receiving AMPH therapy at screening (2, placebo; 39, LDX); mean AMPH dose was 35.0 and 34.1 mg/d for participants in placebo and all LDX groups, respectively. Of the 41 participants, 36 remained symptomatic (ADHD-RS-IV >18) at screening despite receiving AMPH. For the 36 participants in the placebo (n = 2) and LDX (n = 34) groups, respectively, at endpoint, mean change from screening ADHD-RS-IV total scores were -5.5 and -14.8 and from baseline scores were -13.5 and -17.8. For the overall study population, endpoint mean change from baseline ADHD-RS-IV total scores were -7.8 for placebo and -17.5 for LDX. In the prior AMPH subgroup, 2/2 (100.0%) in the placebo group and 22/39 (56.4%) participants in the LDX (all doses) group reported any TEAE. Events that occurred in ≥5% for LDX were dry mouth (5/39; 12.8%), headache (5/39; 12.8%), fatigue (3/39; 7.7%), insomnia (3/39; 7.7%), decreased appetite (2/39; 5.1%), and nausea (2/39; 5.1%). None of these events occurred in the 2 placebo patients with prior AMPH use.

**Conclusion:**

In these post hoc analyses, adults with significant baseline ADHD symptoms despite adequate AMPH treatment dose showed similar improvements in ADHD symptoms with LDX treatment as the overall study population. Prospective studies are needed to confirm these findings. The safety profile of LDX in the overall study population was consistent with long-acting psychostimulant use.

**Trial registry:**

Study to Assess the Safety and Efficacy of NRP104 in Adults With Attention-Deficit Hyperactivity Disorder (ADHD). Clinicaltrials.gov Identifier: NCT00334880

## Background

Attention-deficit/hyperactivity disorder (ADHD) affects approximately 4.4% of adults in the United States [1]. Currently, psychostimulants, both amphetamines (AMPH) and methylphenidate (MPH), are considered first-line ADHD pharmacotherapy in adults [2-4]. Clinically nonresponsive patients treated with one psychostimulant will often obtain an improved clinical response when switched to another psychostimulant class; possibly because classes may differ in mechanisms of action [5]. As reviewed by Arnold and colleagues, AMPH and MPH are reuptake inhibitors of norepinephrine and dopamine (DA) [5]. However, AMPH also stimulates neurotransmitter release from presynaptic receptors.

Moreover, it is observed in psychiatry that, when patients exhibit efficacy or tolerability concerns while on a treatment, it is not uncommon in clinical practice (e.g., depression treatment) for a patient to be switched to a different medication within the same class [6,7]. In ADHD treatment and management, this has included switching from an immediate-release or short-acting formulation to a long-acting formulation [8,9], or from a racemic mixture to a single enantiomer formulation (e.g., d-MPH) [10].

The AMPH class of psychostimulants comprises short- and long-acting formulations of mixed AMPH salts (MAS) and MAS extended release (MAS XR), respectively, and d-AMPH formulations, including short-acting d-AMPH and long-acting d-AMPH (d-AMPH-ER), and the prodrug lisdexamfetamine dimesylate (LDX). LDX is a long-acting prodrug stimulant indicated for ADHD treatment in children (6 to 12 years), adolescents (13 to 17 years), and adults [11]. The inactive prodrug is converted, primarily in the blood, to l-lysine and therapeutically active d-AMPH [12]. MAS XR is a once-daily beaded formulation, also indicated for ADHD treatment in children (6 to 12 years), adolescents (13 to 17 years), and adults [13]. Overall, studies have demonstrated the effectiveness and safety of LDX and MAS XR in adults with ADHD [14-18]. However, a qualitative comparison in a matched-group post hoc analysis suggested that LDX provided greater improvement in ADHD symptoms [16]. Unlike LDX, the pharmacokinetic profile of MAS XR is altered by gastrointestinal pH variations, as assessed by coadministration with a proton pump inhibitor that reduces stomach acid [19].

A PubMed literature search of relevant papers produced none that systematically assessed treatment response in patients switched between different formulations of the same class of stimulant (e.g., from one MPH or AMPH formulation to another). Therefore, a study assessing treatment effects of a more recent psychostimulant formulation in adults who remain symptomatic while using another psychostimulant formulation may be useful to clinicians choosing between treatment options for adults with ADHD.

The objectives of these post hoc analyses were to assess the efficacy of LDX in adults with ADHD who remained symptomatic on AMPH therapy (various formulations) prior to enrollment vs the overall study population in a 4-week, placebo-controlled, LDX trial. Comparing symptomatic recent and prior AMPH users to the overall study population of adults with ADHD treated with LDX can aid clinicians in determining if LDX may be a viable treatment option after another AMPH medication has been used with suboptimal treatment response.

## Methods

### Study design and participants

This was a multicenter, randomized, double-blind, forced-dose titration, 4-week study that evaluated efficacy of LDX (30, 50, and 70 mg/d) vs placebo in adults (18 to 55 years) with ADHD. The study was performed in accordance with the Declaration of Helsinki and the International Conference on Harmonisation Guidelines for Good Clinical Practice. All participants provided written informed consent and Institutional Review Board approval was obtained at all study sites prior to study conduct. Institutional Review Boards/Ethics Committees approving the study were Aspire IRB, 9320 Fuerte Dr, Suite 105, La Mesa, CA 91941 (multiple study sites); Duke University Medical Center IRB, Hock Plaza, 2424 Erwin Rd, Suite 405, Durham, NC 27705; Office of Institutional Review (UHC IRB) University Hospitals of Cleveland, 11100 Euclid Ave, Lakeside 1400, Cleveland, OH 44106-7061; Partners Human Research Committee, 116 Huntington Ave, Suite 1002, Boston, MA 02116; Subcommittee for Human Studies (SHS), 423 E 23rd St, 18S, New York, NY 10010; UCSF Committee on Human Research, 3333 California St, San Francisco, CA 94143-0310; University of California, Irvine, Institutional Review Board, Office of Research Administration, 300 University Tower, Irvine, CA 92697; Western Institutional Review Board (WIRB), 3535 Seventh Ave, SW, Olympia, WA 98502; and Yale University School of Medicine Human Investigation Committee (HIC), 47 College St, Suite 208, PO Box 208010, New Haven, CT 06520. The methodology and results of the primary and secondary analyses from this study have been reported elsewhere [20]. The study included participants on AMPH formulations, including MAS, MAS XR, and d-AMP (d-AMPH spansule and short-acting d-AMPH) at screening and who, at screening, remained symptomatic on their prior treatment. Post hoc analyses of these participants were conducted and are reported here. Participants may have previously been on more than one type of AMPH.

Key inclusion criteria included a primary ADHD diagnosis, by *Diagnostic and Statistical Manual of Mental Disorders, Fourth Edition, Text Revision*[21] (DSM-IV-TR) criteria and an ADHD Rating Scale IV [22] (ADHD-RS-IV) with adult prompts [23] total score of ≥28 at baseline.

### Primary efficacy assessments

The primary efficacy measure was the ADHD-RS-IV with adult prompts that assessed ADHD symptoms at screening, baseline (after washout of prior treatment), and endpoint. Endpoint was defined as the last postrandomization treatment week with a valid ADHD-RS-IV score. The primary efficacy endpoint was the mean change from baseline to endpoint in ADHD-RS-IV total score in the overall efficacy or intention-to-treat (ITT) population (all randomized and treated participants who had a baseline ADHD-RS-IV score and at least one postrandomization ADHD-RS-IV score).

### Other efficacy measures

The Clinical Global Impressions [24] (CGI) scale evaluated global ADHD symptom illness severity and improvement. CGI-Severity (CGI-S) was assessed at baseline using a 7-point scale with scores ranging from 1 (normal, not at all ill) to 7 (among the most extremely ill)*.* The CGI-Improvement (CGI-I) scale was assessed at each postbaseline visit also on a 7-point scale with scores ranging from 1 (very much improved) to 7 (very much worse).

### Post hoc assessments

The prior AMPH subgroup was defined as all participants who took AMPH products with a stop date on or after the screening date. For purposes of this post hoc analysis, an ADHD-RS-IV total score >18 at screening in the prior AMPH subgroup was considered a suboptimal level of symptom control. ADHD symptom items are rated on a 4-point scale, which consists of scores of 0 (never or rarely), 1 (sometimes), 2 (often), and 3 (very often) [22]. An ADHD-RS-IV total score of ≤18 (an average score of 1 per item for the 18-item scale) has been used to define symptomatic remission in combined-type ADHD [25]. As reviewed by Steele and colleagues, when individuals are treated (with or without medication), symptomatic remission in ADHD should be defined as a “loss of diagnostic status, minimal or no symptoms, and optimal functioning” [26]. Moreover, on most standardized questionnaires, symptomatic remission can be “operationalized as a mean total score of ≤1” for each item or an ADHD-RS-IV total score of ≤18.

### Safety assessments

Safety assessments in the overall safety population included treatment-emergent adverse events (TEAEs), vital signs, laboratory findings, and electrocardiogram (ECG).

### Statistical analysis

Descriptive statistics were used to assess efficacy outcomes in the prior AMPH subgroup. No comparative statistical analyses were conducted between the prior AMPH-treated subgroup and the overall study population since the study was not prospectively designed to assess these comparisons. Based on established criteria [26,27], clinical response was defined as a change in ADHD-RS-IV total score of ≥30% from baseline and a CGI-I score of 1 or 2. Symptomatic remission, a measure of optimal symptom control, was defined as a postbaseline ADHD-RS-IV total score ≤18.

The overall safety population comprised all participants enrolled and randomized who received treatment. TEAEs were defined as events with onset postrandomization (i.e., first treatment date).

## Results

### Disposition

Of 420 participants randomized, 414 (62 receiving placebo, 352 LDX) were included in the efficacy population, 71 of 420 (16.9%) were discontinued, and 41 of 414 (9.9%) were receiving AMPH at screening. Of the 41 prior AMPH-treated participants, 2 were randomized to placebo and 39 were randomized to LDX (30 mg/d [n = 11], 50 mg/d [n = 16], and 70 mg/d [n = 12]). In the placebo group, 1 participant was treated with MAS and 1 with MAS XR. In the LDX (all doses) group, 11 participants were previously treated with MAS, 27 with MAS XR, and 2 with d-AMPH. Thirty-six (87.8%) of 41 participants remained symptomatic (ADHD-RS-IV >18) at screening.

### Demographics and baseline characteristics (Table 1)

**Table 1:** Demographics and baseline characteristics of the prior AMPH subgroup (n = 41) and overall safety population (n = 420)

**Variable, n (%)**		**Prior AMPH subgroup**		**Overall safety population**	
		**Placebo**	**LDX (all doses)**	**Placebo**	**LDX (all doses)**
		**n = 2**	**n = 39**	**n = 62**	**n = 358**
**Sex**	Male	0	20 (51.3)	32 (51.6)	196 (54.7)
	Female	2 (100.0)	19 (48.7)	30 (48.4)	162 (45.3)
**Race**	White	1 (50.0)	38 (97.4)	48 (77.4)	301 (84.1)
	Non-white	1 (50.0)	1 (2.6)	14 (22.6)	57(15.9)
**Ethnicity**	Hispanic/Latino	0	1 (2.6)	6 (9.7)	34 (9.5)
	Non-Hispanic/Non-Latino	2 (100.0)	38 (97.4)	56 (90.3)	324 (90.5)
**CGI-S at baseline**	Moderately ill	1 (50.0)	12 (30.8)	27 (43.5)	115 (32.1)
	Markedly ill	1 (50.0)	17 (43.6)	25 (40.3)	195 (54.5)
	Severely/extremely ill	0	10 (25.6)	10 (16.1)	48 (13.4)

The mean (standard deviation [SD]) ADHD-RS-IV total score at screening for the prior AMPH subgroup was 39.3 (7.0) for placebo and 41.0 (5.7) for LDX. Mean (SD) AMPH doses were 35.0 (7.1) mg/d for those randomized to the placebo group and 30.0 (8.9), 38.4 (17.7), and 32.2 (22.2) mg/d for those randomized to the 30-, 50-, and 70-mg/d LDX groups, respectively. Moreover, duration of prior AMPH exposure was reported in the range of approximately 2 weeks to 13 years; only 1 participant was treated for <4 weeks. Two patients reported MAS doses of <20 mg/d.

### Primary efficacy measures

**Figure 1:**
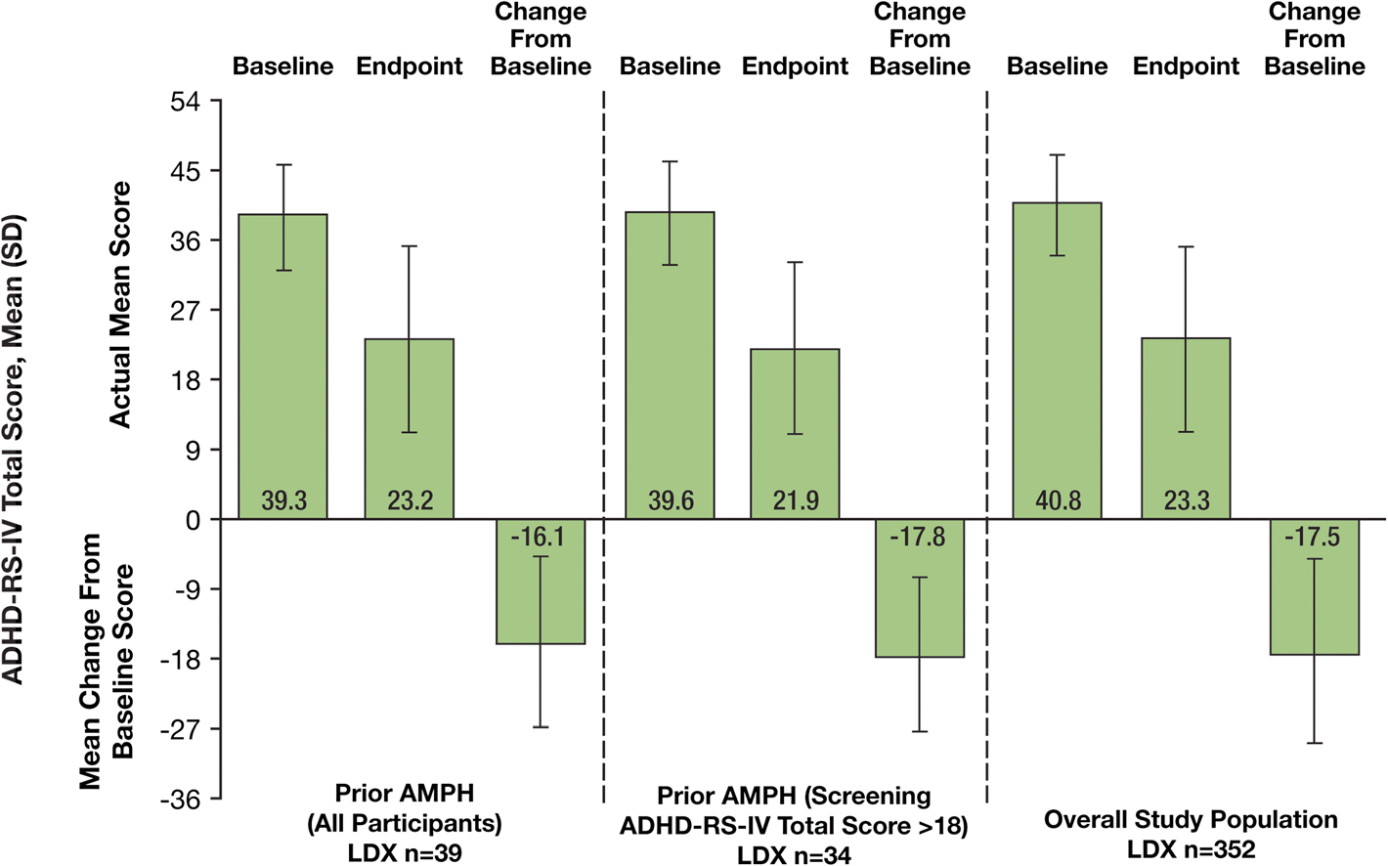
Mean (SD) ADHD-RS-IV total scores for prior AMPH LDX-treated subgroup and overall efficacy population.

At endpoint, change from baseline in mean (SD) ADHD-RS-IV total scores for LDX-treated participants was similar in AMPH groups and the overall study groups (Figure 1). Prior AMPH nonresponders (ADHD-RS-IV total score >18 at screening) in the placebo group (n = 2) had baseline mean (SD) ADHD-RS-IV total score of 41.0 (5.66) and change from baseline was -13.5 (4.95). In the placebo group of the overall efficacy population (n = 62), baseline mean (SD) ADHD-RS-IV total score was 39.4 (6.42) and change from baseline was -7.8 (9.28). Because of the small number of participants in the placebo-treated prior AMPH nonresponders group (n = 2), no comparisons between placebo and LDX AMPH nonresponders subgroups were warranted.

### Other efficacy measures

**Figure 2:**
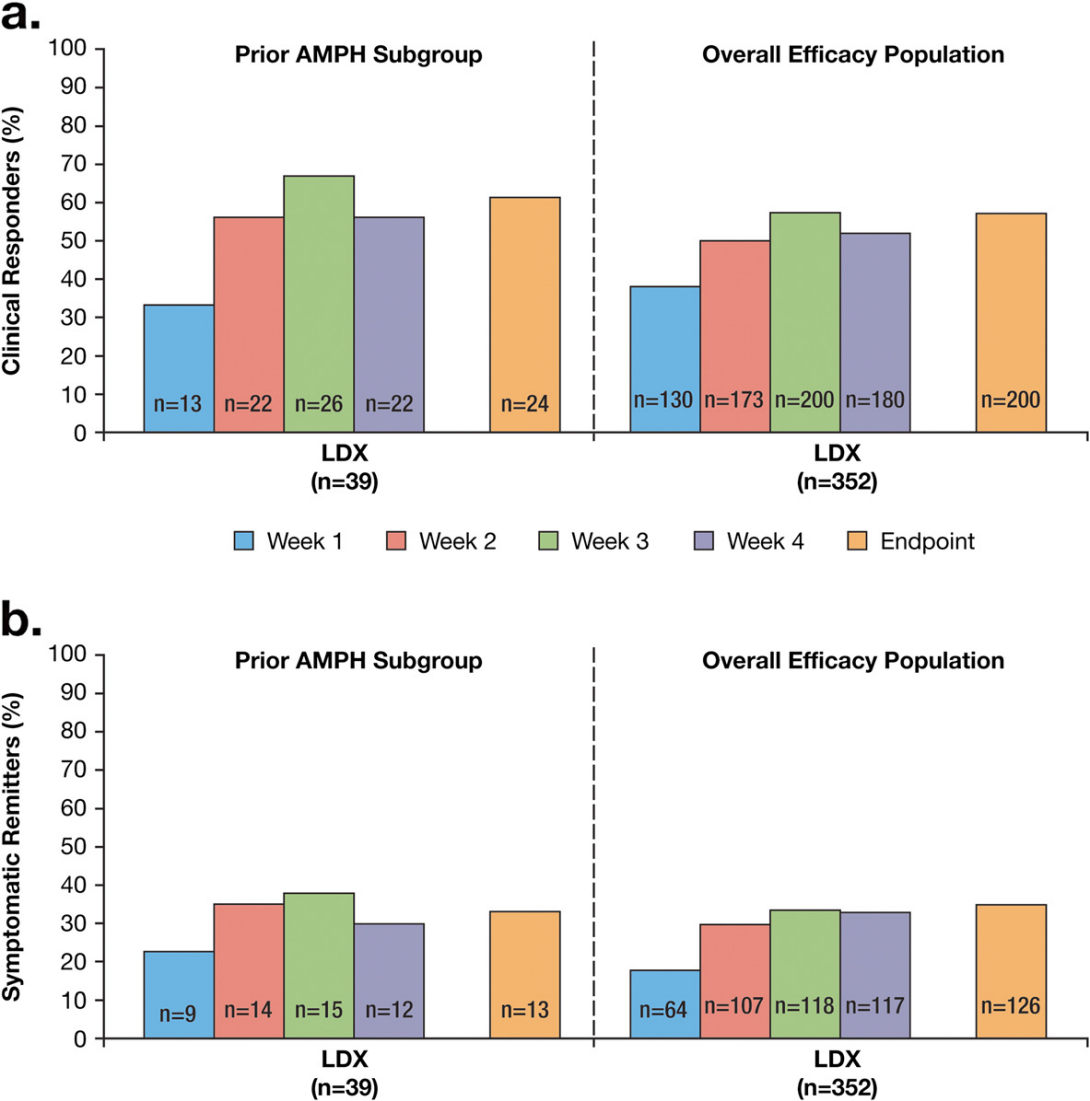
**Percentage of a) clinical responders and b) symptomatic remitters.** Note: Clinical response was defined as a change in ADHD-RS-IV total score of ≥30% from baseline and a CGI-I score of 1 or 2. Symptomatic Remission was defined as a post baseline ADHD-RS-IV total score of ≤18.

The mean (SD) CGI scores were comparable between the prior AMPH subgroup and overall efficacy population in the LDX-treated groups (Table 2). At all time points assessed, the percentage of clinical responders and symptomatic remitters was comparable in both LDX groups (Figures 2a and 2b). For 2 participants in the placebo-treated prior AMPH subgroup, 1 (50.0%) achieved clinical response at week 1 through 4 and at endpoint; and 1 (50.0%) achieved symptomatic remission at week 2 only. For participants in the overall efficacy population receiving placebo (n = 62), the proportion achieving clinical response ranged from 11.3% at week 1 to 27.4% at week 4 and 29.0% at endpoint; the proportion achieving symptomatic remission ranged from 1.6% at week 1 to 11.3% at week 3 and 11.3% at endpoint.

**Table 2:** Mean (SD) CGI-S and CGI-I scores for prior AMPH subgroup and overall efficacy population

**Variable**	**Mean (SD) CGI scores**			
	**Prior AMPH subgroup**		**Overall efficacy population**	
	**Placebo (n = 2)**	**LDX (n = 39)**	**Placebo (n = 62)**	**LDX (n = 352)**
**CGI-S (Baseline)**	4.5 (0.71)	4.9 (0.76)	4.7 (0.73)	4.8 (0.65)
**CGI-I (Endpoint)**	2.5 (0.71)	2.4 (1.11)	3.2 (1.19)	2.4 (1.07)

### Safety

In the prior AMPH subgroup, 2 of 2 (100.0%) in the placebo group and 22 of 39 (56.4%) participants in the LDX (all doses) group reported any TEAE (Table 3). For those receiving LDX in this subgroup, TEAEs with ≥5% frequency were dry mouth (5/39; 12.8%), headache (5/39; 12.8%), fatigue (3/39; 7.7%), insomnia (3/39; 7.7%), decreased appetite (2/39; 5.1%), and nausea (2/39; 5.1%). There were only 2 prior AMPH participants randomized to placebo; therefore, describing “common” TEAEs is not appropriate, but neither placebo participant experienced one of the TEAEs common in the LDX group. All TEAEs in all prior AMPH users were of mild to moderate severity and there were no serious TEAEs.

**Table 3:** Common TEAEs with frequency ≥5% in the LDX (all doses) group and greater than placebo

**Preferred terminology (MedDRA 9.1)**	**Participants, n (%)**			
	**Prior AMPH subgroup**		**Overall safety population**	
	**Placebo n = 2**	**LDX (All Doses)**	**Placebo n = 62**	**LDX (All Doses)**
		**n = 39**		**n = 358**
**All TEAEs**	2 (100)	22 (56.4)	36 (58.1)	282 (78.8)
**Anorexia**	0	0	0	18 (5.0)
**Anxiety**	0	1 (2.6)	0	21 (5.9)
**Decreased appetite**	0	2 (5.1)	1 (1.6)	95 (26.5)
**Diarrhea**	0	0	0	24 (6.7)
**Dry mouth**	0	5 (12.8)	2 (3.2)	92 (25.7)
**Fatigue**	0	3 (7.7)	3 (4.8)	17 (4.7)
**Headache**	0	5 (12.8)	8 (12.9)	74 (20.7)
**Initial insomnia**	0	1 (2.6)	2 (3.2)	18 (5.0)
**Insomnia**	0	3 (7.7)	3 (4.8)	69 (19.3)
**Irritability**	0	1 (2.6)	4 (6.5)	22 (6.1)
**Nausea**	0	2 (5.1)	0	25 (7.0)
**Upper respiratory tract infection**	0	1 (2.6)	3 (4.8)	20 (5.6)

In the overall safety population, 36 of 62 (58.1%) in the placebo group and 282 of 358 (78.8%) participants in the LDX (all doses) group reported any TEAE (Table 3). The majority of TEAEs were mild to moderate in severity. Twenty-two of 420 (5.2%) participants were discontinued due to TEAEs in the overall safety population. In this population, there were no deaths, and 2 of 420 (0.5%) participants had serious AEs (leg injury due to motor vehicle accident [LDX 30 mg/d group] and postoperative knee pain [LDX 70 mg/d group]). Both reported serious AEs were considered not treatment-related, and participants were discontinued.

At endpoint for the overall safety population, small mean increases in vital signs (systolic and diastolic blood pressure) from baseline were not statistically significant vs placebo. At endpoint for pulse, the slight mean difference vs placebo was significant (*P* = .0018). There were no significant meaningful changes in QTcF interval data from baseline between the placebo and LDX groups.

## Discussion

In these post hoc analyses, adults with significant baseline ADHD symptoms in the prior AMPH group, despite adequate mean AMPH treatment dose and duration of prior treatment (only 1 participant was treated for <4 weeks), showed improvements in symptoms with LDX treatment similar to the overall study population. Improvement in ADHD symptoms in LDX-treated adults was similar in the prior AMPH subgroup and overall efficacy population. Moreover, global severity at baseline and global symptom improvement with LDX treatment were comparable across all treatment groups in the prior AMPH subgroup and overall efficacy population.

One study suggests that, although psychostimulants (both AMPH and MPH) are effective in ADHD management, some participants responded better to one type of psychostimulant than to the other [28]. However, results of studies that assess treatment response after switching between agents in the same class are few. A comparative review of psychostimulants suggests that many studies assessing differences between psychostimulants do not show comparisons at the individual participant level [5]. Thus, it is unknown if patient variability in terms of prior treatment history may affect response to current treatment.

This study suggests a differential response to various ADHD formulations within the same class of psychostimulants may occur, as indicated by the improved clinical response with LDX treatment in participants who had significant ADHD symptoms despite prior AMPH therapy. Although conducted in animals with results that may not apply to humans, a study by Joyce et al supports this contention; potential variability in response based on formulation differences among the same AMPH class of psychostimulants was suggested by differential response in AMPH-evoked DA release with MAS (racemic mixture of 76% d-AMPH and 24% l-AMPH salts), d-AMPH, and d, l-AMPH in the rat striatum [29].

Such variations in neurotransmitter release based on the differing formulations of the same class of psychostimulant may play a pivotal role in intrapatient variability to treatment response. LDX, which is a prodrug of d-AMPH covalently bound to therapeutically inactive l-lysine, has demonstrated consistent and low inter-and intrapatient pharmacokinetic variability in d-AMPH mean observed maximum drug concentration and area under the concentration-time curve from time zero to infinity_,_ as well as consistent delivery of d-AMPH in adults [30]. Although a small amount of LDX is hydrolyzed to d-AMPH in the gastrointestinal tract, the conversion into active d-AMPH occurs primarily in the blood. The LDX conversion to d*-*AMPH is unlikely to be affected by gastrointestinal pH and variations in normal gastrointestinal transit times [31,32].

Another open-label, adult study that assessed LDX and MAS XR pharmacokinetics, alone or in combination with omeprazole (proton pump inhibitor) demonstrated that MAS XR-treated participants on omeprazole experienced a shortened time to maximum drug concentration (T_max_) of ≥1 hour in more than 50% of participants vs MAS-XR alone. However, LDX combination therapy with omeprazole resulted in shortened T_max_ in only 25% of participants vs LDX alone [19]. Moreover, the study indicated that the distribution around the median d-AMPH T_max_ for LDX was unaffected by omeprazole administration, although for MAS XR the dispersion was compressed. These aforementioned study data suggest a variable pharmacokinetic response even among the same class of psychostimulants. Data in animal models suggest that amphetamine formulations may differ in their pharmacodynamic effects as well. In rats administered equivalent doses of LDX and d-AMPH, increases in striatal dopamine release and in locomotor activity were lower in peak effect but more sustained with LDX vs d-AMPH [33].

A post hoc comparative qualitative analysis, using groups that were matched based on treatment duration, baseline ADHD symptom severity, and approximately equivalent AMPH doses of LDX and MAS XR in adults with ADHD from 2 similar short-term trials, found that both psychostimulants demonstrated efficacy vs placebo. Safety profiles were consistent with psychostimulant use [16]. However, this qualitative analysis also suggested that LDX treatment vs MAS XR demonstrated greater numerical improvements in ADHD core and global symptoms, as well as decreased frequency of percent differences (active treatment minus placebo) in AEs. Although exploratory in nature, these data suggest that there may be within-class efficacy and safety differences among psychostimulants; however, prospective and quantitative head-to-head comparison trials are needed to confirm these findings.

Findings from clinical trials have not provided clinicians with sufficient comparative data to adequately assess which psychostimulant may be optimal for individual patients, especially since large variations in response rates to drugs and doses exist, and the best sequence of dispensing the various psychostimulant treatments by the clinician is currently unknown [34]. Studies such as the present analysis may prove beneficial to clinicians in determining appropriate treatment options after nonresponse or suboptimal response to a particular psychostimulant therapy.

Overall, the safety profile of LDX was consistent with other long-acting psychostimulants. The frequency of common TEAEs ≥5% appeared to be lower in the LDX-treated prior AMPH groups compared with the overall population, perhaps because these patients were acclimated to the effects of psychostimulant medications. This effect was also seen in a pediatric study of LDX with patient groups that were previously treated with psychostimulants [35].

Limitations of this analysis include results that may not be representative of large cohorts because of the small subgroup sample sizes and the study design features discussed below. Since analyses were not designed or powered to assess group differences and were described with summary statistics, prospective studies are needed to confirm these results. The majority of participants were non-Hispanic/non-Latino, white, and moderately to markedly ill at baseline; results may not be able to be generalized to other ethnicities, races, or global illness severity levels. Due to the post hoc nature of this analysis, factors related to prior use of AMPH were not controlled or assessed in the study: despite the knowledge that adequate mean doses of prior AMPH were used, there was no information to determine if these doses were clinically optimized; data on the level of compliance with prior AMPH treatment were also lacking; and individuals with poor tolerability to AMPH would be ineligible to participate in this study, presenting another study limitation. The baseline symptom severity before AMPH treatment was unspecified. In addition, there was no apparent limitation on study enrollment that would exclude participants with sufficient clinical response to prior medication, since symptomatic nonremitters on prior AMPH were not defined by overall clinical response to AMPH, but only by the participant’s screening ADHD-RS-IV total score.

## Conclusions

Overall, the analyses provide a signal suggesting that, for patients who are not optimally treated with AMPH formulations, LDX remains a potential alternative to consider for the treatment of ADHD in adults. In addition, efficacy outcomes in the prior AMPH subgroup population were consistent with those of the overall study population. The LDX safety profile was consistent with long-acting psychostimulant use. However, this study was not designed to address or compare relative advantages and disadvantages of particular pharmacotherapeutic alternatives. Prospective trials assessing this signal would be helpful in determining the utility of such options in clinical management of patients requiring treatment changes.

## Abbreviations

ADHD: Attention-deficit/hyperactivity disorder; ADHD-RS-IV: ADHD Rating Scale IV; AEs: Adverse events; AMPH: Amphetamine; CGI: Clinical Global Impressions; CGI-I: CGI-Improvement; CGI-S: CGI-Severity; DA: Dopamine; d-AMPH: dextroamphetamine; DSM-IV-TR: Diagnostic and Statistical Manual of Mental Disorders Fourth Edition, Text Revision; ECG: Electrocardiogram; ITT: Intention to treat; LDX: Lisdexamfetamine dimesylate; l-amphetamine: levo-amphetamine; MAS: Mixed AMPH salts; MPH: Methylphenidate; SD: Standard deviation; TEAEs: Treatment-emergent AEs; T_max_: Time to maximum concentration; XR: Extended release.

## Competing interests

Dr Babcock is an employee of Shire and holds stock and/or stock options in Shire.

Dr Dirks is an employee of Shire and holds stock and/or stock options in Johnson & Johnson and Shire.

Mr Adeyi is an employee of Shire and holds stock and/or stock options in Shire.

Dr Scheckner is an employee of Shire and holds stock and/or stock options in Shire.

## Authors’ contributions

**TB** was the associate director, Scientific Publications, Clinical Development and Medical Affairs for this study, and made substantial contributions to the analysis and interpretation of the data. He was deeply involved in drafting the manuscript and revising the intellectual content. He has given final approval of this version. **BD** was the director, Clinical Development and Medical Affairs for this study, and made substantial contributions to the analysis and interpretation of the data. He was deeply involved in drafting the manuscript and revising the intellectual content. He has given final approval of this version. **BA** was a statistician involved in all post hoc data analysis, interpretation, and presentation. Statistician BA was fully involved in drafting and revising the intellectual content of this manuscript. Statistician BA has given final approval to this version. **BS** was a director, Scientific Publications, Clinical Development and Medical Affairs, for this study, and made substantial contributions to the analysis and interpretation of the data. He was deeply involved in drafting the manuscript and revising the intellectual content. He has given final approval of this version.

## Authors’ information

**Thomas Babcock, DO**, is currently an employee of Shire Pharmaceuticals LLC, where he has worked in the Medical Affairs Department since 2005. Dr Babcock graduated from the University of Osteopathic Medicine and Health Sciences (now Des Moines University) in Des Moines, Iowa, and earned his doctorate in anthropology working in Central America. Dr Babcock has authored 12 journal articles and 1 book as of January 2011.

**Bryan Dirks, MD**, is currently a medical director with Shire Pharmaceuticals LLC. Dr Dirks is a diplomate of the American Board of Psychiatry and Neurology in general psychiatry. He also has a master’s of science degree in epidemiology from Harvard University School of Public Health in Boston, Massachusetts, and a master’s of business administration degree from George Washington University in Washington, DC. His research publications include work in suicidology, schizophrenia, and ADHD.

**Ben Adeyi, MS, ACII**, is currently an employee of Shire Pharmaceuticals LLC, and has been working in the Biostatistics and Statistical Programming Department since 2008. He has worked as a senior biometrician for Merck & Co., Inc., and, prior to working in the pharmaceutical industry, he was a senior data analyst in the Nuclear Cardiology Medicine Department at Cornell University Medical College in New York, New York. Mr Adeyi was educated at Emory University in Atlanta, Georgia, and Temple University in Philadelphia, Pennsylvania. He is a charter member of the Chartered Insurance Institute of London, England, and has coauthored several abstracts and manuscripts.

**Brian Scheckner, PharmD, BCPP, CMPP**, is currently an employee of Shire Pharmaceuticals LLC, where he is a Director of Scientific Publications. He has worked for Shire since 2004 in the Clinical Development and Medical Affairs Department, serving in publication and medical communication roles. Dr Scheckner was educated at the University of the Sciences in Philadelphia (PharmD) and Rutgers University (BS, Pharmacy), and has licenses/certifications in pharmacy, psychiatric pharmacy, and publications planning. His membership in professional associations includes the International Society for Medical Publication Professionals (ISMPP) and the College of Psychiatric and Neurologic Pharmacists (CPNP). His research publications include work in ADHD and MDD.

## Pre-publication history

The pre-publication history for this paper can be accessed here:

http://www.biomedcentral.com/2050-6511/13/18/prepub
